# Antibiotic prescriptions among dentists across Norway and the impact of COVID-19 pandemic

**DOI:** 10.1186/s12903-023-03380-6

**Published:** 2023-09-08

**Authors:** Farnoush Tousi, Mohammed Al Haroni, Stein Atle Lie, Bodil Lund

**Affiliations:** 1 Department of Clinical Dentistry, University of Bergen, Bergen, Norway; 2 Department of Maxillofacial Surgery, Haukeland University Hospital, Bergen, Norway; 3 Department of Clinical Dentistry, Faculty of Health Sciences, UiT the Arctic University of Norway, Tromsø, Norway; 4 Centre for New Antimicrobial Strategies, UiT the Arctic University of Norway, Tromsø, Norway; 5 Department of Dental Medicine, Karolinska Institute, Stockholm, Sweden; 6 Medical Unit of Plastic Surgery and Oral and Maxillofacial Surgery, Department for Oral and Maxillofacial Surgery and Jaw Orthopedics, Karolinska University Hospital, Stockholm, Sweden

**Keywords:** Antibiotic prescription, Dentistry, Dentist, Consumption, Defined daily doses (DDD), COVID-19, Phenoxymethyl penicillin, Amoxicillin, Metronidazole, Clindamycin, Amoxicillin, Clavulanic acid, Co-amoxiclav

## Abstract

**Background:**

The prescription of antibiotics in dental practice contributes significantly to the total use of antibiotics in primary healthcare. This study aimed to evaluate antibiotic prescription in dental practice during the years 2016–2021 in Norway and their relative contribution to national outpatient consumption and to investigate the influence of age, gender, geographic region, and COVID-19. A further aim was to review differences in prescribing patterns to verify effect of governmental strategies to reduce over-prescribing of antibiotics.

**Methods:**

This register study investigated the national antibiotic prescription between 2016 and 2021. Data was obtained from the Norwegian prescription register, the Norwegian Institute of Public Health and Statistics Norway. The consumption of 12 common antibiotics was measured using WHO defined daily doses (DDDs), DDD per 1000 inhabitants per day (DIDs _1000_).

**Results:**

A total of 6,049,445 antibiotic prescriptions of the 12 investigated compounds were issued in primary care during the study period. Dentists accounted for 942,350 prescriptions corresponding to 15.6% of the total. An overall decrease in the number of prescriptions by health professions other than dentists during the 5 years (IRR = 0.92, 95% CI:0.92–0.93, *p* < 0.001) was observed. For dentists a slight increase in the number of prescriptions (IRR = 1.01, 95% CI: 1.01–1.01, *p* < 0.001) was seen over the study period. The increase of antibiotic prescriptions in dentistry was more pronounced during the COVID-19 pandemic. The 4 most prescribed type of antibiotics based on average number of DDDs of the total period 2016–2021 were in descending order; phenoxymethylpenicillin (1,109,150) followed by amoxicillin (126,244), clindamycin (72,565), and metronidazole (64,599). An unexpected finding was that the prescription of the combination compound amoxicillin/clavulanic acid had significantly increased in dentistry during the last 5 years. Geographic, gender, and age differences in the rates of prescriptions were also seen. The data revealed that there are seasonal variations in dental prescriptions.

**Conclusions:**

Noticeable differences exist in prescribing patterns of antibiotics in the last 5 years. Restricted access to dental care due to COVID-19 may have resulted in increased antibiotic prescribing in dentistry as opposed to an otherwise downward trend. Despite national guidelines there is still a need for improvement of antibiotic stewardship in dentistry and to define effective methods to disseminate information.

## Background

Awareness of the growing problem of antibiotic resistance has steadily increased over the last two decades discussed in many studies [1–4]. The use of antibiotics became the foundation for the treatment of infectious diseases since their discovery and has helped to save millions of lives [4]. Today, because of antibiotic use, overuse and misuse, antibiotic resistance is on the rise, which subsequently leads to the increase of morbidity, mortality, and treatment costs for infectious diseases [5].

The use of antibiotics is an integral part of practicing dentistry, both to prevent infections in vulnerable patients and to treat ongoing systemic infections of dental origin [2, 6, 7]. Dentoalveolar abscesses with systemic involvement, facial cellulitis, and osteomyelitis are examples of oral infections requiring systemic antibiotic treatment. In most cases, in combination with surgical interventions such as incision, drainage, and/or tooth extraction. Untreated or delayed treatment may in some cases lead to severe infections spreading beyond the oral cavity threatening airways and vital structures [8]. In Norway, the recommendations for treatment for odontogenic infections governed by Norwegian Directorate of Health which recommends phenoxymethylpenicillin (1 g every 6^th^ hour in adults for 5 days) for acute dentoalveolar infections, where there is an indication to use antibiotics, as a first choice. In case of therapeutic failure with the first drug of choice, the addition of metronidazole (400 mg every 8 hourly in adults for 5 days) is recommended. Oral diseases, such as dental caries and periodontal diseases, if not controlled and treated properly, may precede to cause acute infections in underlying tissues. Gram-positive bacteria in supragingival biofilm, such as *Streptococcus* spp., *Lactobacillus* spp., and *Actinomycetes* spp., are associated with the development of dental caries. Gram-negative bacteria in subgingival biofilm, such as *Porphyromonas gingivalis*, *Actinobacillus* spp., *Prevotella* spp., and *Fusobacterium* spp. have been shown to have a role in the development of periodontal diseases. The bacteria in both supragingival and subgingival biofilm have been shown to be up to 1000-fold more resistant to antibiotics compared to bacteria in suspension [9, 10]. Dentists also prescribe antibiotics prophylactically to prevent infection. This can either be due to patients with high risk factors for infection such as pronounced immunosuppression or because the planned procedure is regarded to be associated to unacceptably high risk for infection [11]. When prophylactic antibiotic is indicated, the recommendation in Norway is one dose of 2 g amoxicillin 1 h before invasive procedures.

WHO adopted a global action plan to fight antimicrobial residence (AMR) in 2015 [12]. As a result of this, the Norwegian government published the national strategy against antibiotic resistance in June 2015. In 2016, the Norwegian Ministry of Health and Care Service published an action plan against antibiotic resistance in the health service, with the goal of reducing antibiotic use in the population by 30 percent by the end of 2020 [13]. Furthermore, in 2019, the Ministry of Health published an action plan for optimizing infection control. The actions plan`s main goal is to reduce health service-associated infections and provide a better organization and structure of infection control in Norway. As a result, national recommendations for infection control and hygiene guidelines at the dental health service was implemented, and solid guidelines or recommendations in streamlining the clinicians’ decision process regarding antibiotic use was put in place [1, 14].

Studies have shown that the dentists’ prescriptions accounted for about 8–11% of total national consumptions of antibiotics in Norwegian health care [6, 15]. Inappropriate prescribing of antibiotics in dentistry is reported from several parts of the world [16–19]. In Norway, there is a relatively low consumption of antibiotics, and it is among the three lowest prescribing countries in Europe with prescription predominately of narrow-spectrum antibiotics [15, 20].

There is an interest to know how the Covid-19 pandemic restrictions on dental care services in Norway may have had an impact on antibiotic prescribing by dentists. Because dental care services were partially closed or open with a reduce capacity during the COVID-19 pandemic, many patients chose to postpone regular checks and treatments, and it is anticipated that there might have been an increased risk of compensatory prescription of antibiotics. This is also the case because of the number of telephone consultations also increased [21]. It is necessary to continue to provide, implement, maintain, and follow up the national guidelines by well-structured organization so that antibiotics will continue to be effective for users in the future.

The main objective of this retrospective study was to monitor and evaluate the number of national antibiotic prescriptions written by dentists between 2016 and 2021 in all Norwegian counties. A further objective was to investigate the influence of demographic factors as age, gender, county, and specialty in profession on prescription of antibiotics and review differences in prescribing patterns to verify effect of governmental strategies to reduce over-prescription. A further aim was to describe the impact of the policy to restrict dental access on antibiotic prescribing during COVID-19 pandemic.

## Methods

### Data source

The aggregated data on antibiotic prescription were obtained from the prescription register, the Norwegian Institute of Public Health. The Norwegian Prescriptions Database (NorPD) was established on January 1^st^ in 2004 at the Norwegian Institute of Public health. The NorPD monitors drugs dispensed by Prescriptions in Norway by using an ATC system (Anatomical Therapeutic Chemical classification) and a unit of measurement called the Defined Daily Dose (DDD). Drugs that are purchased without prescriptions over the counter or supplied to hospitals and nursing homes are not included [22]. The retrieved data was received as encrypted and aggregated files and did not include any patient related information such as personal data and the diagnosis indicating the prescription.

Data of the number of inhabitants and the number of practicing dentists in different Norwegian counties per year, were obtained from Statistics Central bureau Norway (SSB).

### Retrieved data

The data material was based on all dispensations of antibiotics for human use in Norway during the period January 1^st^, 2016, to December 31^st^, 2021. The prescriptions from dentists were separately accounted for to allow comparison. The data included 12 antibiotics namely: doxycycline, oxytetracycline, tetracycline, amoxicillin, phenoxymethylpenicillin, co-amoxiclav (amoxicillin and clavulanic acid), erythromycin, spiramycin, clarithromycin, azithromycin, clindamycin, and metronidazole. These antibiotics are the most common antibiotics prescribed in dental practice in Norway [6, 15, 22]. The DDD was given for each prescribed antibiotic compound. The extent of each antibiotic used, related to dentistry-based prescribing, was calculated as the corresponding number of prescriptions per 1000 inhabitants per day (DIDs _1000_).

Number of prescriptions distributed based on patient age groups (0–6 years, 7–19 years, 20–64 years, 65–79 years, and over 80 years) and per quarter in the period 2016–2021, was also retrieved. The data collected regarding the prescribers were region of practice, gender, and year of birth. The distribution of prescriptions between different specialties within odontology was also retrieved and included oral surgery and oral medicine, periodontics, endodontics, pedodontics, oral prosthetics, orthodontics, oral and maxillofacial radiology, and general dentistry.

### Statistical analyses

Regression analysis for the number of prescriptions or defined daily dose (DDD) was performed. For the unadjusted analyses Poisson regressions were applied, while for models with multiple variables, negative binomial regression was used to consider overdispersion in the data. The results are presented as incidence rate rations with 95% confidence intervals, and p-values for the hypotheses of no difference between categories of the variables. All analyses were analyzed using Stata version 17.0 (Stata Inc, TX, USA). P-values less than 0.05 were considered statistically significant.

### Ethical considerations

An approval from NorPD was required prior to onset. A prerequisite for this approval was that no data could be traced back either to prescribers or to patients. Thus, no personal information or personal medical information that could hamper anonymity was retrieved. No ethical approval was required since data was received as aggregated data made available from related official bodies upon approval of project application.

## Results

### Antibiotic prescription

**Fig. 1:**
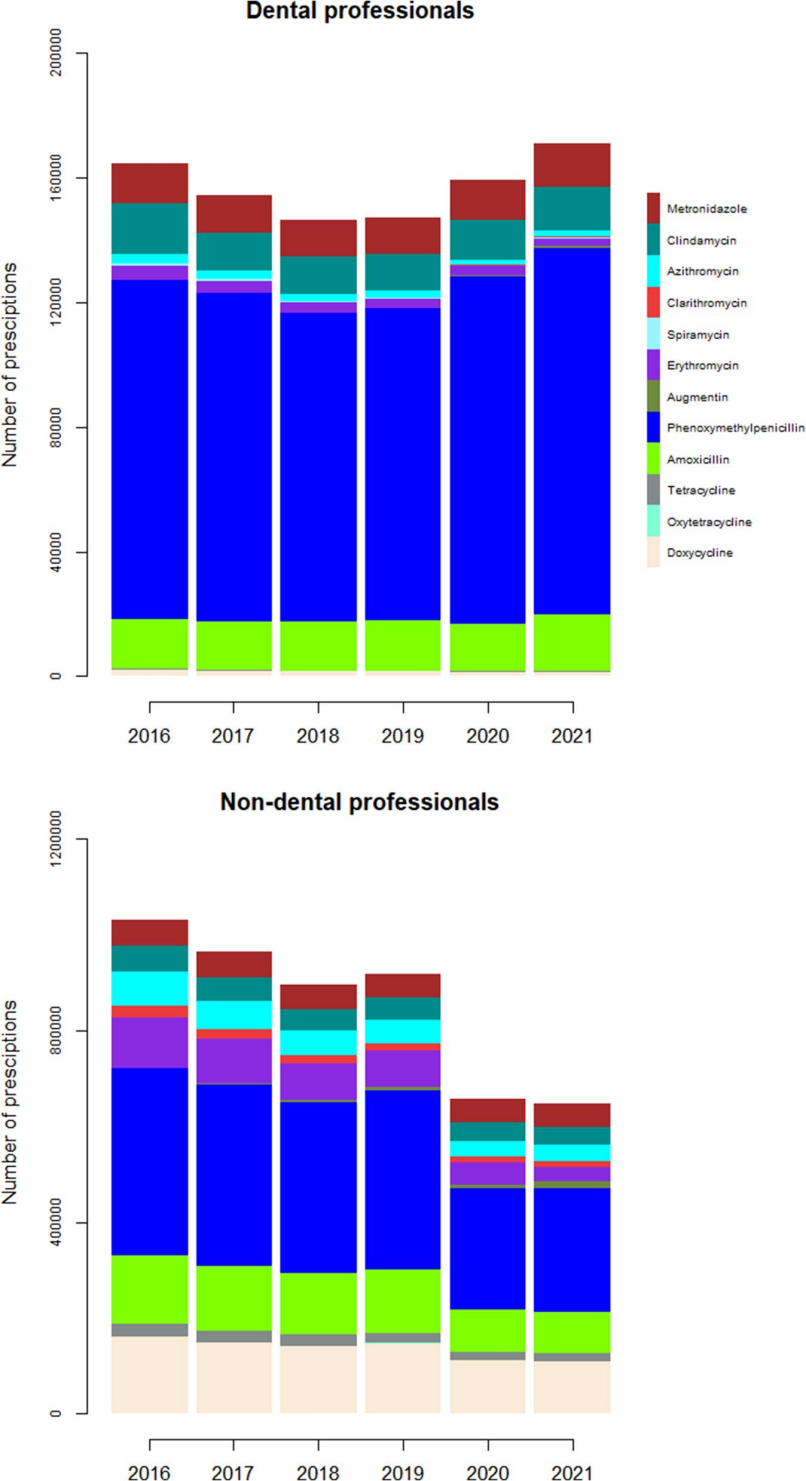
The 12 antibiotics ranked by the number prescriptions by dentists and non- dentists each year respectively

**Fig. 2:**
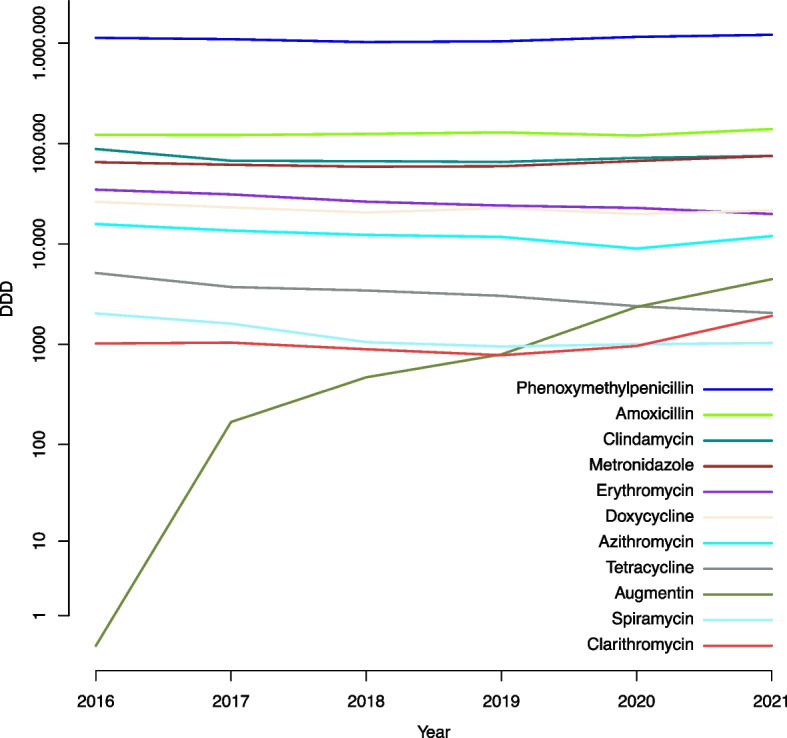
Number of DDDs by dentists each year for the different types of antibiotics

There were 6,049,445 antibiotic prescriptions among dentists and non-dentists for human use in the period 2016–2021. A total of 942,350 prescriptions were issued by dentists in the same period for the 12 antibiotics investigated in the current study. The total antibiotic prescriptions issued by dentists and non-dentists for each year are shown in Table 1. The 12 antibiotics ranked by the number prescriptions in each year by dentists and non-dentists are displayed in Fig. 1. The result showed an overall decrease in the number of prescriptions by non-dentists during the 5 years period (IRR = 0.92, 95% Cl:0.92–0.93, *p* < 0.001). For dentists a slight increase in the number of prescriptions was found (IRR = 1.01, 95% Cl: 1.01–1.01, *p* < 0.001). When comparing the number of prescriptions per year for dentists to the decreased overall number of prescriptions, the relative increase was higher (IRR = 1.09, 95% Cl:1.09–1.09, *p* < 0.001). The number of DDDs by dentists for each year for the different types of antibiotics are shown in Table 2 and Fig. 2. The data were sorted by the highest to the lowest average value of DDDs by dentists for the total period 2016–2021.The average number of DDDs prescribed by dentists in the period 2016–2021, the four most prescribed types of antibiotics were in descending order; phenoxymethylpenicillin (1,109,150), followed by amoxicillin (126,244), clindamycin (72,565), and metronidazole (64,599). Most prescriptions were prescribed by general dentists followed by oral surgeons and periodontologists. The distributions of the number of prescriptions by general dentists and other specialties are shown in Table 3. The increase of co-amoxiclav was significant over the 5 studied years. Co-amoxiclav was prescribed mostly by general dentist followed by oral surgeons and periodontologists as shown in Table 4.

**Table 1:** Total number of prescriptions by dentist and non-dentists each year

Year	2016	2017	2018	2019	2020	2021
Number of prescriptions by dentists	164,509	154,213	146,122	147,048	159,218	171,240
Number of prescriptions by non-dentists	1,194,016	1,116,207	1,040,576	1,064,591	816,324	817,731

**Table 2:** Cross tab, that shows the number of DDDs and percent (%) of total prescriptions by dentists for each year for the different types of antibiotics. The table is sorted by the highest to the lowest average value of DDDs for the total period 2016–2021

ATC	2016		2017		2018		2019		2020		2021			
	DDD^a^	%^b^	DDD^a^	%^b^	DDD^a^	%^b^	DDD^a^	%^b^	DDD^a^	%^b^	DDD^a^	%^b^	Total DDD	Average DDD
Phenoxymethylpenicillin	1128778	66.2%	1095013	68%	1024328	67.7%	1042353	68.2%	1153864	69.9%	1210564	68.7%	6654901	1109150
Amoxicillin	122152	9.7%	121623	10%	124732	10.9%	129260	10.9%	120268	9.6%	139426	10.5%	757461	126244
Clindamycin	88305	9.8%	67408	8%	66805	8.1%	65599	7.9%	71960	8.0%	75315	8.0%	435392	72565
Metronidazole	65294	7.9%	61553	7.9%	58845	8.0%	59467	7.9%	67063	8.1%	75370	8.5%	387592	64599
Erythromycin	34781	2.7%	31170	2.6%	26383	2.2%	24153	2.0%	22863	1.8%	19901	1.4%	159250	26542
Doxycycline	26356	1.2%	23152	1.1%	20578	1.0%	22778	1.0%	19889	0.9%	21634	0.8%	134387	22398
Azithromycin	15818	1.8%	13617	1.7%	12318	1.6%	11764	0.1%	8999	1.0%	11992	1.3%	74508	12418
Tetracycline	5144	0.2%	3731	0.2%	3444	0.2%	3046	0.2%	2404	0.1%	2058	0.1%	19827	3305
Augmentin	0	0.0%	168	0.0%	470	0.0%	795	0.1%	2362	0.2%	4463	0.4%	8258	1376
Spiramycin	2036	0.4%	1610	0.3%	1053	0.2%	953	0.2%	1003	0.2%	1033	0.2%	7689	1282
Clarithromycin	1020	0.1%	1042	0.1%	895	0.1%	780	0.1%	964	0.1%	1925	0.1%	6626	1104
Total	1489683	100%	1420088	100%	1339851	100%	1360948	100%	1471639	100%	1563682	100%	8645891	1440982

^a^Number of DDD

^b^Percent of total prescriptions

**Table 3:** Cross tab, that shows the number of prescriptions prescribed by general dentist and different specialties for each year 2016–2021

Specialties	2016	2017	2018	2019	2020	2021
General practitioner	131,589	125,476	118,653	118816	130039	137957
Oral surgery/Oral medicine	15378	13997	14,486	15530	16050	18518
Periodontics	8882	7622	6549	6350	6179	7421
Endodontics	5850	4920	4537	4753	5320	5630
Prosthetics	1941	1698	1459	1233	1292	1476
Orthodontics	710	366	276	229	247	161
Pedodontics	109	87	124	108	50	35
Oral and maxillofacial radiology	18	14	8	0	0	0

**Table 4:** Cross tab, that shows the number of prescriptions of Augmentin prescribed by general dentists, periodontists, and oral surgeons for each year 2016–2021

Specialty	2017	2018	2019	2020	2021	Total
General practitioner	10	51	49	154	244	508
Oral surgery	0	9	16	74	297	396
Periodontist	11	0	42	101	84	238
Total	21	60	107	329	625	1142

### Influence of age and gender

There were 4,908 prescriptions (0.1%) by dentists for patients aged 0–6, 22,563 prescriptions (2.4%) in the age group 7–19, 667,079 prescriptions (70.6%) in the age group 20–64, 204,237 prescriptions (21.9%) in the age group 65–79, and 43,563 prescriptions (5%) in the age group over 80 years.

The number of dentists registered as prescriber of at least one prescription with the ATC codes by dispensing year and prescriber’s gender were 4817 in 2016, 4847 in 2017, 4895 in 2018, 4908 in 2019, 4996 in 2020, and 5049 in 2021. There was no overall difference in the prescriptions between the different ATC codes over the 5-year period (p = 0.240). A significant difference in number of prescriptions between male and female prescribers was found (IRR = 0.75 95% Cl:0.58–0.96, *p* < 0.028) where female dentists prescribed 25% less antibiotics than that of male dentists. There is a significant difference in prescription based on prescribers’ year of birth. Prescribers with year of birth from 1941 or before accounted for 0.20% of the prescriptions, those born between 1942–1962 accounted for 24.3%, similarly those between 1963–1983 accounted for 54.8%, and those from and later 1984 accounted for 20.6%.

### Geographical differences

**Fig. 3:**
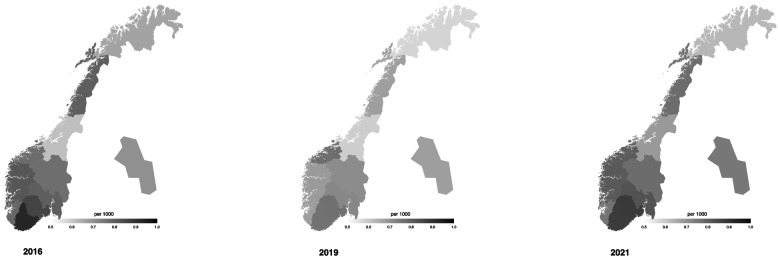
DID by dentists for the different counties in year 2016, 2019, and 2021

There was generally no statistical difference in DID (DDD/1000/year) between the different Norwegian counties adjusted for the number of inhabitants in the respective counties. However, the DID in Trondelag county, and Troms and Finnmark county were significantly lower than that of Agder county in the period 2016–2021. The number of DID and antibiotic prescriptions were markedly lower in Northern counties compared to the Southern part of Norway and the lowest was found in the central part of Norway (Fig. 3).

There was no significant association between age related prescription pattern and county. Hence, the differences found for age groups were the same for all counties. There was also no significant interaction between county and gender, meaning that the difference between genders was the same within the different counties.

### Seasonal prescription pattern

The highest number of prescriptions was in the period October to December (*n* = 243,779). Less prescription was seen in the period July to September (*n* = 222, 648) compared to the average.

## Discussion

Measuring the consumption of antibiotics in any given society is a valuable information to adopt and sustain good polices that enhance reasonable use of antibiotics and preserve their effectiveness in clinical practice. There are six quantity matrices for the outpatient setting (OQM1-6) to measure consumption of antibiotics. Each matric has its advantage and disadvantage [23]. In the current study, only OQM1, OQM4 and OQM6 are provided by the Norwegian prescription database.

Previous international data show that dentists account for about 7–10% of the antibiotics prescribed in primary care [15, 24]. In the current study, the corresponding average rate was 15.6%. Previously, it was reported that the contribution of dental prescriptions to the national prescriptions in Norway between 2010 and 2016 is about 8% [6, 15]. The reported high percentage in the current study could be partly explained by the fact that antibiotic prescription seems to have increased substantially in dentistry during the COVID-19 pandemic. This was probably due to lockdown with strongly reduced availability of dental care. Since Norway is generally considered to be a country with restrictive antibiotic policy the relatively high contribution of dentistry could also be explained by the fact that the reduction of antibiotic prescriptions in the remaining primary care has been very pronounced in the study period [25]. International studies also show significant reduction in total outpatient antibiotic prescriptions issued during the COVID-19 period by physicians and other prescribers [26]. The magnitude of the reduction in the number of antibiotic prescriptions was in children, with respiratory infections [25, 26]. The result of this study shows a relative increase in prescriptions of antibiotics during the COVID-19 pandemic while other international studies show a reduction in antibiotic prescribing among dentists by 8.92% [21]. Since dental care was partially closed during parts of the pandemic period the increasing number of telephone consultations might have contributed to the observed increased in antibiotic prescription. There are studies that raised concerns about the potential for higher antibiotic prescribing rates in virtual consultation than in face- to face communication with the patients [27, 28].

Another finding in the current study is the use of the narrow spectrum phenoxymethylpenicillin was the most common choice among dentists in Norway which is an important core in the country’s conservative prescriptions practice. However, although phenoxymethylpenicillin (Pc-V) represents a dominating proportion of the prescription it would be expected to be even higher since it is the first choice of treatment for dental infections according to Norwegian national recommendations. The only situation in general dentistry requiring clindamycin is in the case of penicillin allergy. Thus, the amount of prescribed clindamycin is not likely to correspond to true type 1 penicillin allergies but could be rather the effect of an overestimation of penicillin allergy and perhaps negligence of the recommendations. In cases of severe odontogenic infections with suspected anaerobic bacteria addition of metronidazole to Pc-V is advocated. Another possible explanation for the relatively high clindamycin prescription could be that it is chosen for these more severe infections. A previous study showed a reduction in the prescription of antibiotics by dentists between 2010 and 2016 by 4.3% [15]. In contrast, this study shows that antibiotic utilization between 2016 and 2021 in dentistry has increased in general and particularly among the main antibiotics used in dentistry in Norway, i.e., Pc-V followed by amoxicillin, clindamycin, and metronidazole.

An unexpected finding was the prescription of co-amoxiclav which had significantly increased among Norwegian dentists during the 5 years study period especially among general dentists. In 2017, the co-amoxiclav compound Augmentin received marketing authorization to be prescribed in Norway. This composite compound contains two different active substances, amoxicillin and clavulanic acid causing more pronounced ecologic disturbance to the normal microbiota compared to the narrow spectrum Pc-V. While amoxicillin is an active against both against Gram-positive and Gram-negative bacteria, clavulanic acid has no antimicrobial effect but binds irreversibly covalent to beta-lactamase [29]. This inactivation of beta-lactamases renders the compound effective against beta-lactamase producing bacteria. The Antibiotic Center for Primary Medicine (ASP) recommends Augmentin in general practice only for treatment infections with *Haemophilus influenza* or *Moraxella catarrhalis* with resistance to amoxicillin, in some cases with chronic obstructive pulmonary disease, and for patients with pneumonia, otitis, or sinusitis. However, according to the Felleskatalog (Encyclopedia of pharmaceutical preparations marketed in Norway), Augmentin is also used for skin and soft tissue infections, including dental infections, infections in bones and joints. The question arises whether the increase in prescription of Augmentin is due to differences in susceptibility among oral bacteria with becomes more resistance that led to treatment failure with conventional antibiotics, or attitudes toward antibiotic prescribing influenced by dentists coming to Norway from abroad, or because of differences in educational guidelines. Before 2021 the recommendations in Norway to use a 600 mg Pc-V dose four times daily for the treatment of dental infections. This dose may have been too low considering that these infections has both soft and hard tissue engagement. Insufficient effect of the recommended antibiotic treatment could also have been a driver for the choice of more broad-spectrum alternatives. To reverse the trend of prescribing broad-spectrum antibiotics, arrangement of audits in antibiotic prescription for dental and oral infections, especially among these who graduated from outside of Norway should be considered by the health authorities.

The results that the population in Agder county of Norway received most antibiotics in Norway, while Trondelag, Troms and Finnmark were significantly lower is in accordance with a previous study [30]. The reason for this finding is not completely clear. It has been suggested that socioeconomic factors could have an impact on antibiotic prescription [31–33]. Statistics provided by NAV (the Norwegian Labour and welfare administration) indicated that Agder has some of the highest level of unemployment in 2021 compared to Trondelag, Troms, and Finnmark. Interestingly, also in Sweden the highest prescription of antibiotics in dentistry is seen in the most southern part of the country [24].

Less prescription was seen from July through September. This could be explained by the fact that dental offices have limited opening hours during this period or because of patients postpone their dental appointments during this period because of summer vacation [34]. Female dentists significantly prescribed fewer antibiotics than that of male dentists, which has been shown in other studies as well [30]. There are studies that shows gender gap in compliance with public policy rules [35]. The results also show that younger dentists prescribe fewer antibiotics than their older counterparts. It seems that the younger dentists are more restrictive and following guidelines updates, more carefully than otherwise older generations. It seems that preservation of antibiotics, sustainability issues and impact on future generations are issues that young people tend to me more aware of than older generations.

## Conclusion

There were marked differences in patterns of consumption of antibiotic attributed to dental prescriptions in last 5 years. It seems that the impact of COVID -19 pandemic, probably because reduced access to dental care, resulted in the increased use of antibiotic prescription compared to an otherwise downward trend. Reduced access to dental service is an important factor which can lead to unnecessary over prescribing of antibiotics. It highlights the essential role of good oral health and availability of dental care in the global efforts to manage antibiotic resistances. The trend of increasing broad-spectrum prescription does not seem to have an apparent association to the pandemic and may be an early warning sign of undesired trends in antibiotic prescription in dentistry. Health authorities are advised to arrange a series of audits in antibiotic prescription for dental and oral infections, especially among these who graduated from outside of Norway.

## Data Availability

The datasets used and analyzed during the current study are available from the corresponding author on reasonable request.

## Abbreviations

AMR: Antimicrobial resistance
NorPD: Norwegian prescription database
ATC: Anatomical therapeutic chemical classification system
DDD: Defined daily dose
DIDs _1000_: DDD per 1000 inhabitants per day
WHO: World health organization
ASP: The antibiotic center for primary medicine
Felleskatalog: Encyclopedia of pharmaceutical preparations marketed in Norway
NAV: Norwegian labor and welfare administration
SSB: Statistics central bureau Norway
Pc-V: Phenoxymethylpenicillin
Co-amoxiclav: Amoxicillin/clavulanic acid
